# Brain computer interface to enhance episodic memory in human participants

**DOI:** 10.3389/fnhum.2014.01055

**Published:** 2015-01-20

**Authors:** John F. Burke, Maxwell B. Merkow, Joshua Jacobs, Michael J. Kahana, Kareem A. Zaghloul

**Affiliations:** ^1^Department of Psychology, Perelman School of Medicine, University of PennsylvaniaPhiladelphia, PA, USA; ^2^Department of Neurosurgery, University of PennsylvaniaPhiladelphia, PA, USA; ^3^Department of Biomedical Engineering, Columbia UniversityNew York, NY, USA; ^4^Department of Psychology, University of PennsylvaniaPhiladelphia, PA, USA; ^5^Surgical Neurology Branch, National Institute of Neurological Disorders and Stroke, National Institutes of HealthBethesda, MD, USA

**Keywords:** BCI, episodic memory, ECoG, theta

## Abstract

Recent research has revealed that neural oscillations in the theta (4–8 Hz) and alpha (9–14 Hz) bands are predictive of future success in memory encoding. Because these signals occur before the presentation of an upcoming stimulus, they are considered stimulus-independent in that they correlate with enhanced memory encoding independent of the item being encoded. Thus, such stimulus-independent activity has important implications for the neural mechanisms underlying episodic memory as well as the development of cognitive neural prosthetics. Here, we developed a brain computer interface (BCI) to test the ability of such pre-stimulus activity to modulate subsequent memory encoding. We recorded intracranial electroencephalography (iEEG) in neurosurgical patients as they performed a free recall memory task, and detected iEEG theta and alpha oscillations that correlated with optimal memory encoding. We then used these detected oscillatory changes to trigger the presentation of items in the free recall task. We found that item presentation contingent upon the presence of pre-stimulus theta and alpha oscillations modulated memory performance in more sessions than expected by chance. Our results suggest that an electrophysiological signal may be causally linked to a specific behavioral condition, and contingent stimulus presentation has the potential to modulate human memory encoding.

## 1. Introduction

In the laboratory setting, episodic memory is commonly studied by presenting participants with a list of items and then asking them to later recall those items. For over a century (Ebbinghaus, 1913), analysis of behavioral data from these tasks has highlighted many intriguing facets of the memory system (Kahana, 2012). Recently, the ability to record electrophysiological data from participants engaging in a memory task has begun to reveal the neural correlates of these behavioral phenomena. While important electrophysiological hallmarks of encoding and retrieval are evident in the time domain (Rugg and Wilding, 2000), many lines of evidence have suggested that neural oscillations have a unique functional role in the memory system (Basar et al., 1999; Kahana, 2006; Nyhus and Curran, 2010).

In particular, data from electroencephalography (EEG) (Klimesch et al., 1997), magnetoencephalography (Osipova et al., 2006), and electrocorticography (ECoG) (Fell et al., 2001; Sederberg et al., 2007) have revealed that changes in theta (4–8 Hz) and alpha (10–14 Hz) oscillations correlate with successful episodic memory encoding and retrieval. In terms of spatial specificity, theta activity has been most commonly observed in the medial temporal lobe and the prefrontal cortex (Nyhus and Curran, 2010; Lega et al., 2011). Indeed, the degree of theta synchronization between these structures has been shown to predict the degree of memory formation (Fell et al., 2003; Anderson et al., 2010; Burke et al., 2013). Similarly, alpha activity has been shown to play a role in memory processing, although the precise direction and meaning of such alpha activity is less certain (Hanslmayr et al., 2012; Waldhauser et al., 2012). Nonetheless, studies using transcranial magnetic stimulation have suggested that such alpha activity also synchronizates across large regions of cortex during memory processing (Zanto et al., 2011).

These theta and alpha electrophysiological correlates of episodic memory have traditionally been observed after stimulus presentation, and are thus interpreted to reflect the act of forming or retrieving a memory. Recent research has extended these findings to the time interval *before* stimulus presentation. Specifically, spectral activity in the theta and alpha frequency bands has been reported to increase prior to successful memory encoding (Guderian et al., 2009; Rutishauser et al., 2010; Fell et al., 2011; Hanslmayr et al., 2013; Merkow et al., 2014) and retrieval (Addante et al., 2011). The observation that on-going neural activity can predict future memory performance is consistent with observations from both scalp EEG and functional imaging studies (Adcock et al., 2006; Otten et al., 2006; Gruber and Otten, 2010; Park and Rugg, 2010). More specifically, although human surface recordings have suggested that theta power in particular is elevated before successful encoding (Guderian et al., 2009; Rutishauser et al., 2010; Hanslmayr et al., 2013), human intracranial recordings from the medial temporal love have also consistently identified an alpha component to this pre-stimulus activity (Fell et al., 2011; Merkow et al., 2014). Thus, empirically, there is data to support that both theta and alpha oscillations play a role in human pre-stimulus memory processing.

These results place episodic memory into the larger context of higher order cognitive functions that are enhanced by ongoing oscillatory activity (Linkenkaer-Hansen et al., 2004; Wyart and Tallon-Baudry, 2009). The functional role of such oscillations in relation to the cognitive event of interest remains speculative; possible mechanisms include increased attention (Driver and Frith, 2000; van Boxtel and Böcker, 2004), phase reorganization to optimize encoding or retrieval (Hasselmo et al., 2002; Hasselmo and Eichenbaum, 2005), or the evolution of temporal context (Polyn et al., 2005; Manning et al., 2011). It is clear, however, that pre-stimulus oscillations, especially in the theta and alpha frequency bands, are correlated with a heightened ability to both encode and retrieve memories. Therefore, if devices could be designed to induce these signals, it may be possible to selectively enhance the episodic memory system (Serruya and Kahana, 2008).

Before devices can be engineered using these pre-stimulus signals, however, it is necessary to establish their causal role, if any, during memory encoding. In particular, the presence of an oscillation before the successful encoding of an episodic memory does not necessarily suggest that inducing that oscillation will enhance successful encoding. Brain computer interface (BCI) experimental paradigms offer an attractive methodology to test this set of issues. Using real-time feedback, a particular electrophysiological event of interest can be used to trigger the presentation of an item to be remembered, and the corresponding behavioral response can subsequently be investigated (Jarosiewicz et al., 2008; Legenstein et al., 2010; Berger et al., 2011). This reverses the traditional dependent and independent variables of the experiment: instead of analyzing electrophysiological correlates of memory, we can analyze the mnemonic correlates of electrophysiology. If the neural oscillation plays a mechanistic role in memory encoding, then a modulation of the electrophysiology should cause an analogous modulation of the behavioral response. Studies using this BCI approach have established that pre-stimulus theta oscillations in the rabbit hippocampus are sufficient to double the learning rate in an associative learning task (Seager et al., 2002; Griffin et al., 2004). Here, we implement a similar approach in humans participants to investigate the role of pre-stimulus theta oscillations in episodic memory.

## 2. Materials and methods

### 2.1. Participants

Participants with medication-resistant epilepsy underwent a surgical procedure in which grid, strip, and depth electrodes were implanted for seizure localization. Data were collected at Thomas Jefferson University Hospital and the Hospital of the University of Pennsylvania. Our research protocol was approved by the institutional review board at each hospital and informed consent was obtained from the participants and their guardians. Our final participant pool consisted of 14 patients (5 Female) left-language dominant). Language dominance was assessed by either the patients' handedness, a clinically administered intracarotid injection of sodium amobarbital (Wada test), or fMRI using a verb generation task (Thomas Jefferson Hospital).

### 2.2. Recordings

Clinical indications alone determined electrode number and placement. Subdural (grids and strips) and depth contacts were spaced 10 mm and 8 mm apart, respectively. Depth electrodes are placed using a frameless stereotactic approach. We recorded intracranial EEG (iEEG) using a Nicolet, Grass Telefactor, or Nihon-Kohden EEG system. Depending on the amplifier and the discretion of the clinical team, the signals were sampled at 400 Hz (Grass), 512 Hz (Nicolet) 500 Hz, 1000 Hz, or 2000 Hz (Nihon Khoden). Signals were referenced to a common contact placed subcutaneously, on the scalp, or on the mastoid process. The testing laptop sent ±5 V analog pulses via an optical isolator into a pair of open lines on the clinical recording system to synchronize the electrophysiological recordings with behavioral events.

For *post-hoc* analyses, all recorded traces were resampled at 256 Hz, and a fourth order 2 Hz stopband butterworth notch filter was applied at 60 Hz to eliminate electrical line noise. In addition, signals were converted to a bipolar montage by differencing the signals between each pair of immediately adjacent contacts on grid, strip, and depth electrodes. We defined the bipolar montage in our data-set based on the geometry of ECoG electrode arrangements. For every grid, strip and depth probe, we isolated all pairs of contacts that were positioned immediately adjacent to one another; bipolar signals were then found by differencing the signals between each pair of immediately adjacent contacts (Anderson et al., 2010). The resulting bipolar signals were treated as new virtual electrodes (henceforth referred to as *electrodes* throughout the text), originating from the mid-point between each contact pair (Burke et al., 2013). All subsequent analyses were performed using these derived bipolar signals.

Contact localization was accomplished by co-registering the post-op CTs with the post-op MRIs using both FSL Brain Extraction Tool (BET) and FLIRT software packages and mapped to both MNI and Talairach space using an indirect stereotactic technique and OsiriX Imaging Software DICOM viewer package. Pre-op MRI's were used when post-op MR images were were not available.

### 2.3. Free recall task

#### 2.3.1. Standard version

Each patient participated in both standard and BCI versions of a free recall episodic memory task. Tasks were administered at the patient's bedside using the python experimental programming language (PyEPL) (Geller et al., 2007). In each version, participants were shown lists of common nouns chosen at random and without replacement from a pool of high-frequency nouns. Each word was visually presented to the patient on a laptop computer screen placed at an arm's length from the patient. Each experimental session of the standard version contained up to 20 lists, and each list contained 15 words. At the start of each trial, a plus sign appeared at the center of the screen to alert patients to the upcoming word presentation and to encourage them to fixate on the center of the screen. The plus sign appeared for 1600 ms, followed by an inter-stimulus interval (ISI). The length of the ISI depended on each version of the task. In the standard version of the task, the ISI was 800 ms followed by a randomly jittered 0 to 400 ms blank interval. The random ISI served to decorrelate physiological responses from successive word presentations. In the BCI version of the task, lists were composed of 10 words that also appeared on the screen for 1600 ms. However, in the BCI version of the task, the length of the ISI depended on the electrophysiologic data (see below).

Immediately after each list presentation, patients were given a series of simple arithmetic problems. This end-of-list distractor task served to reduce the large advantage accorded to end-of-list items during recall (Howard and Kahana, 1999). Each problem took the form of *A* + *B* + *C* = ??, where *A*, *B*, and *C* were randomly chosen positive integers from the set one through nine. After patients solved arithmetic problems for at least 20 s, we presented a row of asterisks accompanied by a 300 ms tone signaling the start of the recall period. Patients were given 45 s to recall list items in any order (standard free-recall instructions). After each session, vocal responses, digitally recorded during the trial, were scored for analysis. Words recalled from the most recent list were considered correct recalls.

#### 2.3.2. Brain computer interface version

In the BCI version of the task, the timing of word presentation depended on the detection of a pre-determined neural oscillation captured from an intracranial contact. To control for variability in ISI's, each experimental session was composed of 20 lists of words divided into two blocks of ten. Lists were composed of 10 study items. In the first block, lists randomly alternated between a contingent condition and a control condition. In the contingent condition (half the lists in the block), presentation of words, and hence the ISI, were contingent on whether a calculated index of power exceeded a pre-determined high threshold. We recorded all ISIs used in the contingent condition, and the first list in the block was always a contingent condition. In the control condition (the remaining half of the lists in the first block), we presented words using the same sequence of ISIs used in one of the contingent conditions, regardless of neuronal oscillatory activity. By using an identical ISI sequence, equal amounts of time are allocated for stimulus encoding in both conditions. In the second block of the contingent condition, we used an identical procedure for determining ISIs, but in this case word presentation during the contingent condition was determined by whether the calculated index of power decreased below a pre-determined low threshold. We compared behavioral performance between the high and low contingent conditions and each condition's respective control condition. Thus, items in the contingent condition were presented based on the amount of power in during the 600 ms preceding each item. In contrast, the items in the control condition, items were presented at random periods with respect to the on-going electrophysiological activity.

### 2.4. Brain computer interface

**Figure 1:**
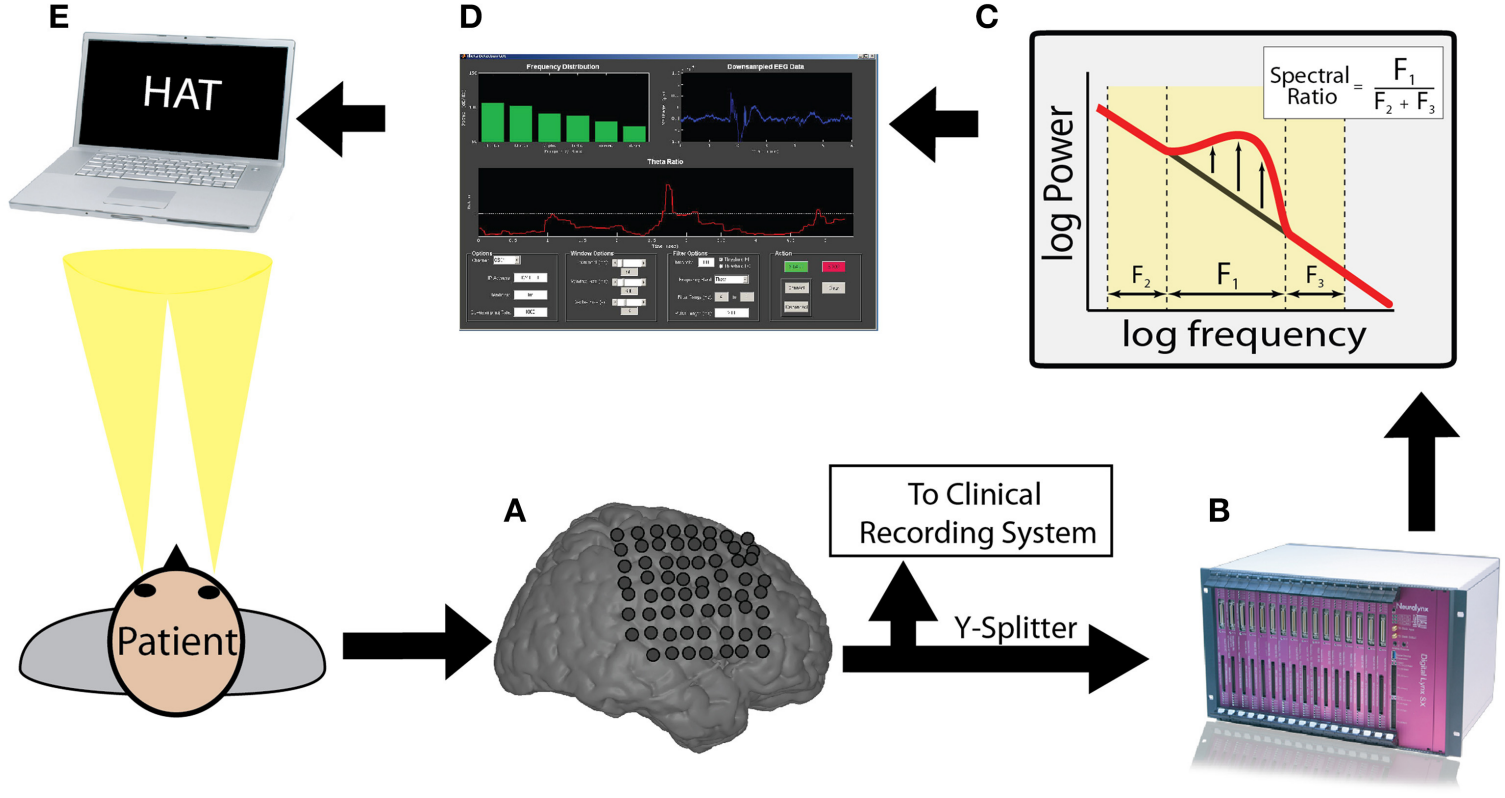
**Brain computer interface free recall task: overview**. Incoming ECoG data recorded by intracranial electrodes **(A)** was split and digitized by a Neuralynx recording system **(B)**. The appropriate memory signal was decoded **(C)** in real-time **(D)** to control the memory experiment **(E)**. The entire real-time loop **(A–E)** was performed within 50 ms.

The closed loop experimental procedure used to present oscillatory contingent word items is shown in Figure 1. We used a Y-splitter to provide a copy of the recorded iEEG signals (Figure 1A) to a research recording system (Neuralynx, Inc. Digital Lynx data acquisition system, Bozeman, MT; Figure 1B). We amplified iEEG signals, sampled at 32 kHz, and bandpass filtered between 0.3 and 300 Hz. We temporarily stored the iEEG signal in a Matlab® (The Mathworks, Inc., Natick, MA) readable buffer using the MatCom software package (Neuralynx, Inc.) before it was written to disk to enable real-time data processing. Data from each electrode stored in this buffer was immediately downsampled to 256 Hz and then used to update and fill a 600 ms sliding window every 50 ms. We extracted theta or alpha oscillatory power from this 600 ms window using a Fast-Fourier Transform (Figure 1C; see Results). We visualized the iEEG signal from the chosen electrode, and the resulting calculated index of power, in real-time using a custom Matlab GUI (Figure 1D). To calculate an index of oscillatory power, we normalized the power in the frequency band of interest by dividing power in this band over the power of equal bandwidths immediately above and below the frequency bandwidth of interest (Seager et al., 2002). The calculated index of power was used to determine the timing of word presentation during the BCI version of the free recall task (Figure 1E).

### 2.5. Data analysis and spectral power

Each participant performed a standard version of the task first in order to quantify memory related changes in spectral power and to identify an optimal oscillation to be used to trigger word presentations in the subsequent BCI version. Downsampled bipolar iEEG signals captured during the standard version of the task were convolved with complex valued Morlet wavelets (wavelet number 7) to obtain magnitude and phase information (Addison, 2002). We used 50 wavelets with center frequencies logarithmically spaced between 2 Hz and 100 Hz. We convolved each wavelet with 3500 ms of iEEG data surrounding each word presentation, from 1000 ms before word onset to 2500 ms after word onset (a 1000 ms buffer was included on both sides of the clipped data). We squared and log-transformed the magnitude of the continuous time wavelet transform to generate a continuous measure of instantaneous power. We averaged these continuous power spectra into two time intervals: a pre-stimulus window (1000–0 ms before word presentation) and a post-stimulus window (300–1500 ms after word presentation).

We then *z*-transformed power values separately for each session using the mean and standard deviation of each electrode's power values sampled every 60 ± 10 s throughout the duration of the session (Burke et al., 2014b). This method allowed us to estimate the mean and standard deviation of each session separately, and corrected for any changes in impedance that occurred during that session.

To assess memory related changes in spectral power within theta (4–8 Hz) and alpha (10–14 Hz) frequencies, we averaged the instantaneous power across each time epoch, and calculated the average power separately across theta and alpha frequencies. For every electrode and for every temporal epoch, we assessed the difference in *z*-scored spectral power in the theta and alpha frequency bands during memory formation by calculating a *t*-statistic on the distribution of power values during successful and unsuccessful encoding. We averaged these *t*-statistics across electrodes for each patient. To generate a *p*-value for changes in spectral power across patients, we performed a one sample *t*-test comparing these across patient distributions to zero (Long et al., 2014).

We also identified the electrodes that exhibited the most reliable change in theta and alpha power between successful and unsuccessful encoding for each participant and for each experimental session of the standard task. Specifically, we calculated a *t*-statistic (and a *p*-value) on the distribution of power values during successful and unsuccessful encoding as above, however we focused on the pre-stimulus window for this analysis. We selected the most reliable electrode and frequency band to use in the contingent condition (as measured by the *p*-value), with the stipulation that the *p*-value must be below the 0.05 level to be used as a trigger during the BCI version of the task.

Once the electrode and frequency band were selected, we ran the BCI version of the task. To determine if the oscillatory contingent presentation of study items significantly modulated memory performance, we compared the rate of correct recall between the contingent and control conditions for both the high and the low trigger conditions using a χ^2^-test. The χ^2^-test was generated using a 2 × 2 table with trigger and control blocks as rows, and number of recalled and number of not recalled words as columns. We determined whether an individual session demonstrated a significant modulation of memory performance by identifying sessions that exhibited a significant difference in recall rate between the contingent and control conditions (*p* < 0.05). To correct for multiple comparisons across sessions, we use a false discovery rate procedure at the *Q* = 0.10 level (Genovese et al., 2002). Specifically, each of the 29 experimental BCI sessions in this study provided two *p*-values using the chi-square tests, the *p*-values for the low and the high-trigger conditions (Table 1). We applied the FDR correction across all of these statistical tests to correct for multiple comparisons.

**Table 1:**
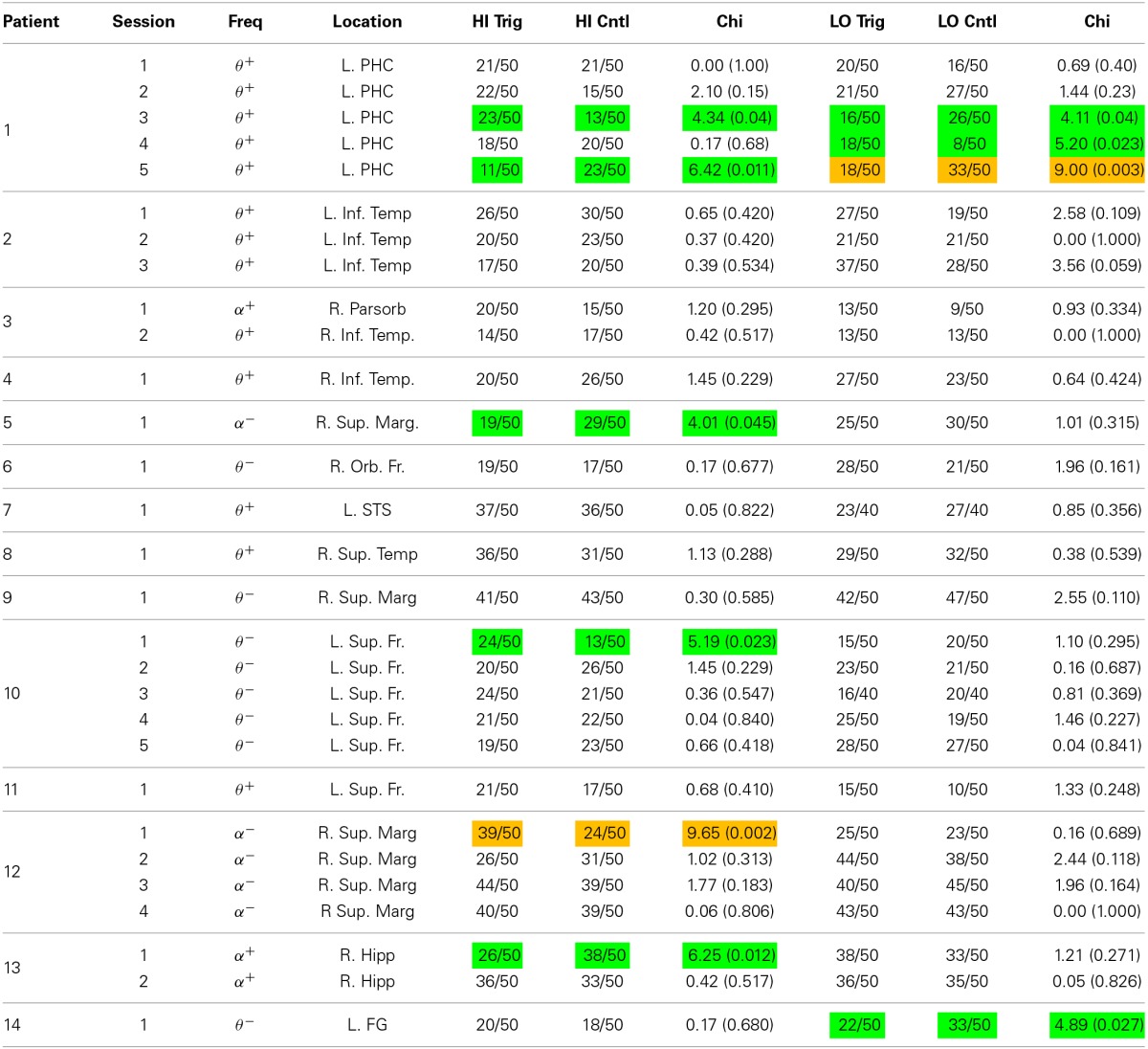
**Results of the bciFR task**.

In the table, the results of the bciFR task are shown for each session from each patient. The number of correctly recalled words (out of the total number of words) is displayed for the high trigger blocks (HI Trig) and the low trigger blocks (LO Trig), as well as for the associated control conditions (HI Cntl and LO Cntl). The χ^*2*^ statistic is shown (Chi), which tested whether the frequency of the recalled words and the not-recalled words differ from one another in the trigger and control conditions. The green boxes represent sessions that displayed modulation of behavioral performance (p < 0.05), and the orange boxes represent sessions that survived correction for multiple comparisons (FDR q = 0.10). Freq, frequency band used in the bciFR task (either alpha or theta). The + and − indicate whether the pre-stimulus effect was an increase or a decrease in power during the pre-stimulus interval. L, Left; R, Right; PHC, parahippocampal cortex; Hipp, hippocampus; Temp, temporal; Inf, inferior; Sup, Superior; Parsorb, parsorbitalis; Marg, Marginal; Orb Fr, Orbital Frontal; STS, Superior Temporal Sulcus; FG, Fusiform Gyrus.

## 3. Results

Fourteen neurosurgical patients with medication resistant epilepsy underwent a surgical procedure in which intracranial electrodes were placed for seizure monitoring. After the surgery, the patients were monitored outside of the operating room with the intracranial electrodes in place for a period of 1–3 weeks. During this period, the participants agreed to run in two different tasks: a standard version of free recall (stFR) followed by an oscillatory contingent, or brain computer interface (BCI), version (bciFR; see Materials and Methods). In both versions of the task, participants were instructed to study a list of words and were then asked to freely recall as many words as possible. However, the amount of time between successive word presentations, or the interstimulus interval (ISI), differed in each version of the task. In the stFR task, the ISI was set at a fixed interval of 800 ms with a 400 ms uniformly distributed jitter. In the bciFR task, the ISI was determined by the amount of spectral power recorded from one of the patient's intracranial electrodes (Figure 1). We selected the electrode and the spectral frequency band based on the data collected in the stFR task.

The stFR task is part of a much larger, multi-center study that has been reported on previously (Manning et al., 2011; Burke et al., 2013, 2014a; Merkow et al., 2014). Here, however, we only report on the subset of patients that also participated in the bciFR task; the BCI data are completely novel data and have not been previously reported in any study.

Behaviorally, during the stFR task, the 14 participants studied 701.8 ± 382.2 words over 46.8 ± 25.5 lists, and successfully recalled 27.6 ± 7.6% of all words (all values represent across patient averages and standard deviations).

**Figure 2:**
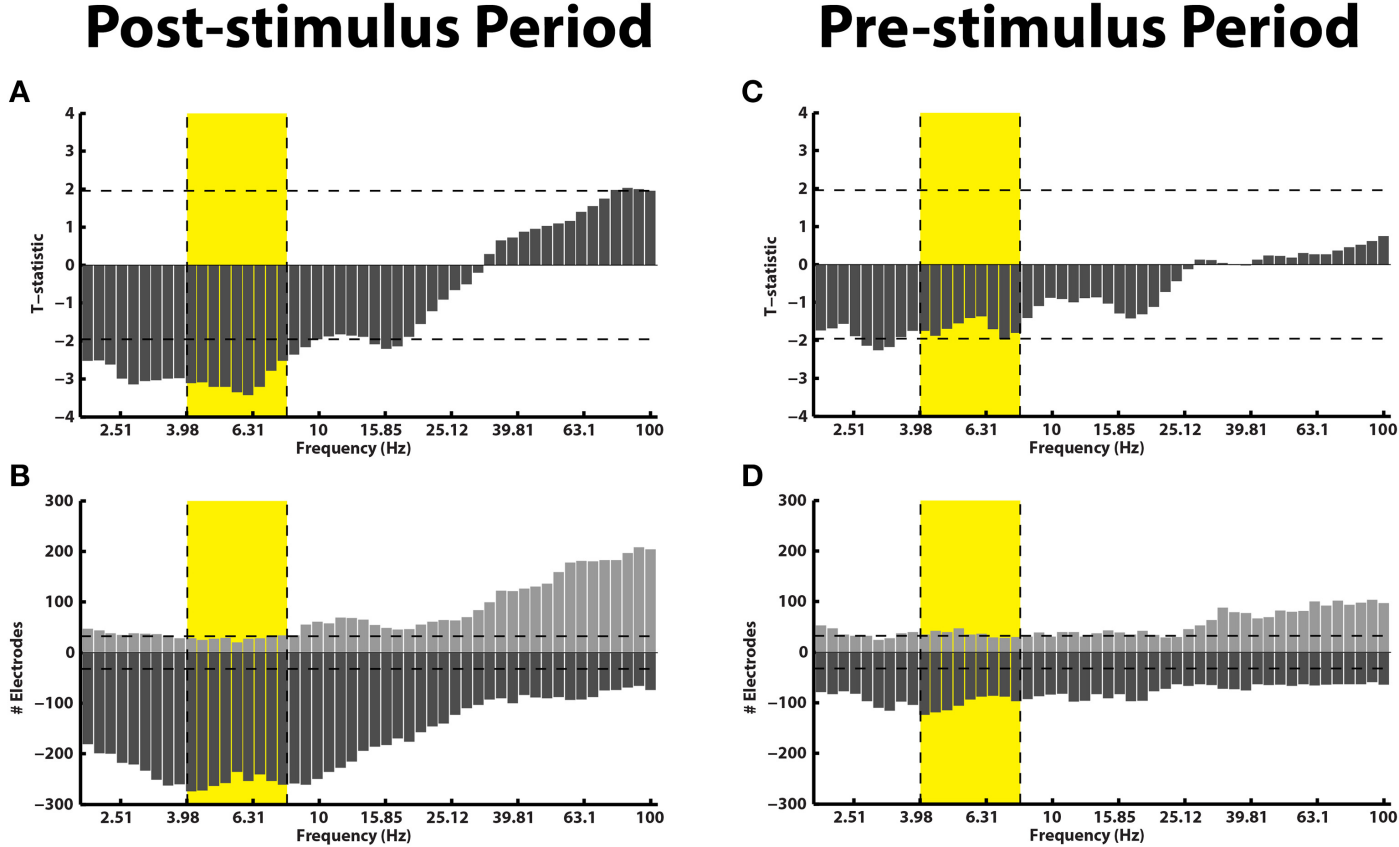
**Subsequent memory effect pre- and post-stimulus**. **(A)** Across all patients, *t*-statistics (y-axis) comparing spectral power during the presentation of items that were later recalled vs. those that were later not-recalled are plotted for all frequencies (x-axis) for the post-stimulus interval (300–1500 ms after word onset). A positive *t*-statistic represents more power in the recalled vs. the not-recalled condition across all 14 patients. The yellow box marks the theta frequency range. The horizontal lines mark the *p* = 0.05 significance level. **(B)** The histogram displays the number of electrodes that showed a statistically reliable (*p* < 0.05) modulation of power during the post-stimulus interval. The horizontal line shows the number of electrodes that should be expected to be significant by chance at the *p* = 0.05 level. **Panels (C,D)** show identical plots for the pre-stimulus interval (0–1000 ms).

Electrophysiologically, we separated the stFR task in two time windows: the post-stimulus and the pre-stimulus intervals. In the post-stimulus time interval (300–1500 ms after word presentation), we calculated spectral power values for all word presentation periods, for each frequency, electrode, and patient (see Materials and Methods). We compared these power values for words that were subsequently recalled and words that were not recalled (Paller and Wagner, 2002). We found that, independent of anatomical location, successful encoding is associated with an overall increase in high-frequency activity and a decrease in low-frequency activity (Figure 2A). This result actually reflects a highly dynamic modulation of spectral power that occurs during successful encoding, which was shown using a larger number of patients in the stFR task (Burke et al., 2014a). Next, we showed this effect by counting the number of electrodes showing a modulation of spectral power (*p* < 0.05) during successful encoding.

The results in Figures 2A,B are consistent with previous reports, and show that the memory results from the subset of patients in this dataset generalize to the overall population (Burke et al., 2014a; Long et al., 2014). However, we were primarily interested in the pre-stimulus interval (0–1000 ms before word presentation), because activity from the pre-stimulus interval could be used to drive the BCI version of the task. In Figures 2C,D, we repeated the analyses described above for the pre-stimulus interval. We find that, first, there is not an overall modulation of spectral power that correlated with successful encoding in the pre-stimulus interval (Figure 2C). Second, although there was not an overall effect across all patients and brain regions in the pre-stimulus interval, we did find that there were a few electrodes that showed a significant modulation of theta/alpha power (Figure 2D). Finally, we performed the analysis in Figure 2 separately for various anatomical regions (see Supplementary Material).

**Figure 3:**
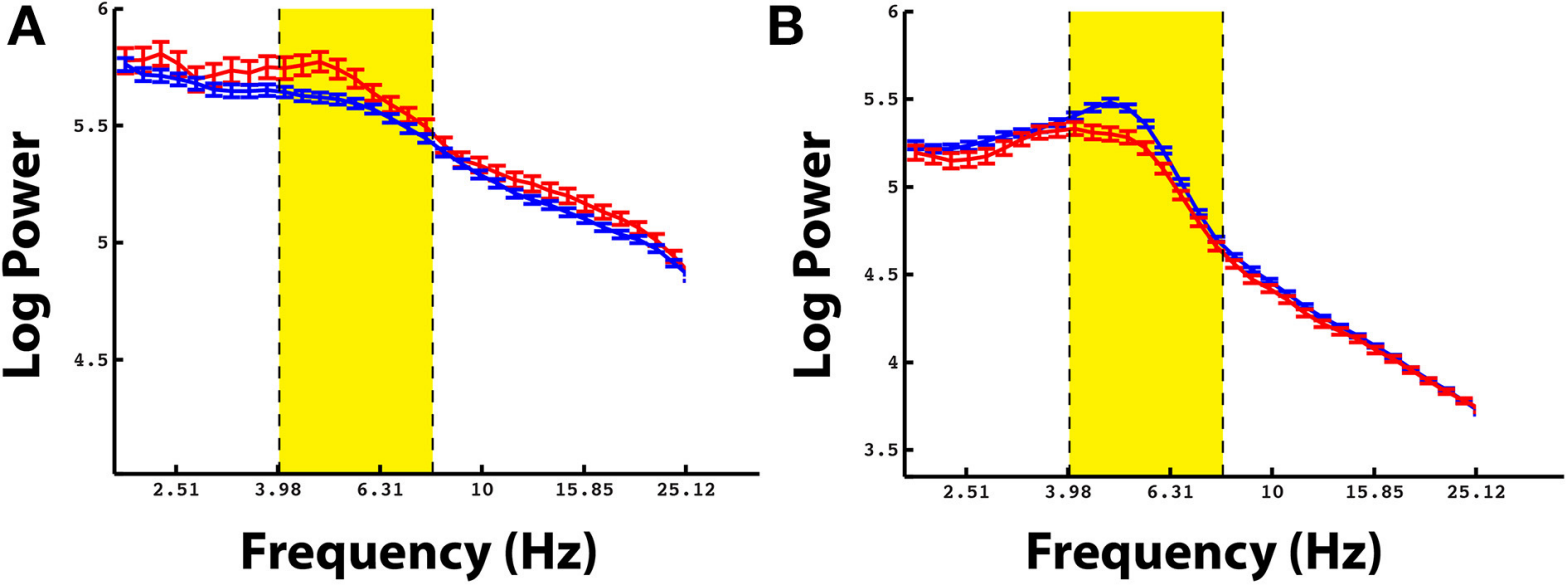
**Example electrodes showing changes in theta during the pre-stimulus time interval**. **Panels (A,B)** show example electrodes in two different patients that displayed marked modulations of theta power in the pre-stimulus interval during successful encoding. The electrodes were taken from the rostral mid-frontal region and the superior frontal region, respectively. The errorbars reflect standard errors on the mean, and the red and blue lines represent power during successful and unsuccessful encoding.

Even though there was not a reliable uni-variate spectral modulation in the pre-stimulus interval that correlated with successful memory encoding, Figure 2D shows that there were certain electrodes that showed a modulation of theta/alpha power during the pre-stimulus period. We therefore used the stFR task to isolate individual electrodes that displayed the most reliable modulation of theta/alpha power in the pre-stimulus interval. Figure 3 displays two such examples, one of which shows an overall positive theta effect (more theta power during successful encoding; Figure 3A) and the other of which shows an overall negative theta effect (less theta power during successful encoding; Figure 3B).

Our goal was to identify if these individual pre-stimulus electrode fluctuations could be used to modulate memory performance. To do that, we identified an electrode in each participant that exhibited the largest difference (*t*-statistic) in theta or alpha oscillatory power between successful and unsuccessful encoding during the pre-stimulus period. Table 1 gives a list of the patients who participated in the bciFR task, and the electrodes (including the location) for each patient that we identified as the most reliable increases in theta/alpha power for each patient. The table lists whether the theta or the alpha band was the most reliable pre-stimulus modulation and the direction of the effect.

Having identified a pre-stimulus marker for memory encoding, participants next performed the bciFR task in which we integrated real-time data acquisition and analysis into the experiment (Figure 1). During the task, we acquired iEEG signals in real-time from the identified electrode and stored this signal in a 600 ms sliding window, updated every 50 ms. We calculated an index of oscillatory power in the identified frequency band of interest by comparing the ratio of the power within the identified band to the power in adjacent frequency bands (see Materials and Methods) (Seager et al., 2002). We used this index to trigger subsequent word presentation in the oscillatory contingent condition of the task. In one block of each experimental session, we triggered word presentation when this index exceeded a pre-determined threshold during the contingent condition (see Materials and Methods). In the second block of each session, we triggered word presentation when this index decreased below this threshold. During control conditions in each experimental block, we used the recorded ISIs during the contingent condition to present word items, regardless of oscillatory power recorded in that electrode. This controlled for the variable timing between word presentations.

**Figure 4:**
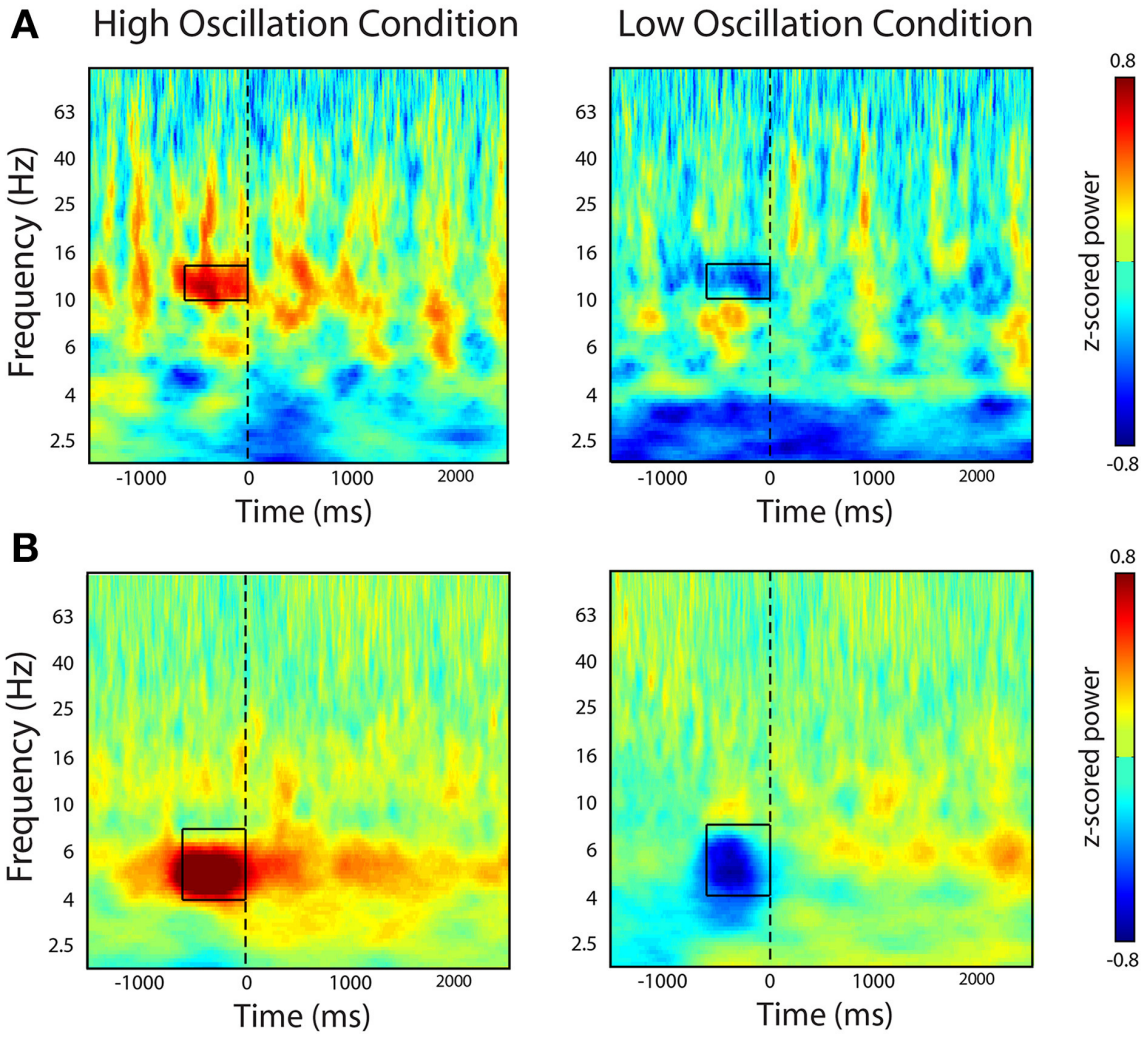
***Post-hoc* time-frequency analysis of spectral power during contingent conditions**. The figure shows time-frequency power spectra averaged across all word presentations during the bciFR task. **(A)** In one participant, we selectively triggered word presentation on increases (left-panel) or decreases (right-panel) of an alpha oscillation. **(B)** In a second participant, we triggered word presentation on increases (left-panel) or decreases (right-panel) of a theta oscillation. Word presentation is indicated by the dashed line at *t* = 0. Color represent average *z*-scored power at every time point and every frequency for all word presentations during the contingent condition.

To confirm that our real-time system triggered word presentation only during the presence of the identified oscillatory marker in the contingent conditions, we examined the average *z*-scored oscillatory power during all word presentations in the contingent condition. In one participant, we triggered word presentation off of alpha oscillatory power (Patient 12 in Table 1). We used this marker to trigger word presentation during the contingent conditions of the BCI version of the task. *Post-hoc* analysis of the average oscillatory power in the contingent conditions indeed revealed that word presentation was preceded by increases or decreases in alpha oscillatory power when increases or decreases, respectively, of the calculated index of power were used to trigger word presentation (Figure 4A). In a second participant, we identified an electrode that demonstrated a modulation of theta oscillatory power during the pre-stimulus encoding period in the standard version of the task (Patient 10; Table 1). Similarly, *post-hoc* analysis of the average *z*-scored oscillatory power surrounding word presentation during the contingent conditions revealed increases or decreases in theta oscillatory power preceding word presentation in the contingent conditions of each experimental block (Figure 4B).

If modulations of pre-stimulus theta and alpha oscillatory activity identified in the standard version of the task are causally related to memory, then we hypothesized that words presented during the presence of these oscillations should be remembered more frequently than words presented at random times. We captured behavioral data from 29 sessions of the BCI version of the task across 14 participants.

Our goal was to modulate memory performance in all patients by first recording activity that correlated with memory encoding, and then use that activity to trigger the presentation of words in a BCI task. Upon implementation, the BCI that we constructed did not modulate memory performance in every patient. However, we did find that the number of experimental sessions in which we elicited a reliable difference (*p* < 0.05; χ^2^-test; see Materials and Methods) in memory performance using the contingent presentation of stimuli was significantly greater than the number of sessions expected by chance (Table 1). In the table, the orange boxes represent four sessions that displayed modulation of behavioral performance after correcting for multiple comparisons using an FDR correction (*q* = 0.10). The green boxes represents a session that trended toward significance (*p* < 0.05), but did not survive multiple comparison correction. In total, 10 sessions showed modulation of memory performance at the *p* = 0.05 level, which was more than expected by chance at this significance level. In summary, although our results did not reliably demonstrate improvement in memory performance, they do support the claim that pre-stimulus iEEG confers information about the memory encoding state in some subjects.

## 4. Discussion

Neural oscillations have been hypothesized to play a mechanistic role in episodic memory formation, however the link between oscillations and memory formation has been largely established by correlational studies. In such studies, memory performance is recorded and used to partition electrophysiological activity into high and low mnemonic states; then the activity in each state is compared to find oscillations that co-vary with memory function. Using this approach, theta/alpha activity in the pre-stimulus interval has been linked to memory formation. If such activity plays a mechanistic role in memory formation, then it could be induced to give an individual a “memory boost” for an arbitrary set of items. For example, an elderly patient could give themselves a *boost* before they encode where they parked their car, or when their physician tells them how to use their medications. Such technology would have a major impact on the ability of a patient with pathological memory loss to perform activities of daily living.

A key step in the development of such technology is to investigate whether pre-stimulus theta/alpha activity plays a mechanistic role in memory function, or whether such activity is a mere epiphenomenon. In order to accomplish this first step, we constructed the BCI in Figure 1 to poll for theta/alpha oscillations in real-time and link them to an episodic memory task. We found that pre-stimulus theta/alpha oscillations were able to boost memory encoding reliably in a limited number of patients/sessions. The fact that a few sessions were successfully modulated by spectral activity in the theta/alpha bands is an important proof-of-principle that, in select cases, the BCI approach to enhance memory formation is feasible. Of note, 6/10 sessions that exhibited a modulation of memory performance (*p* < 0.05) were triggered off of contacts in the medial temporal lobe (MTL; Table 1). More research is needed to assess whether the MTL has a greater capacity for memory modulation then other regions.

Across all patients, the effect was too variable to be implemented as a mnemonic device. Understanding and reducing this variability represents the main hurdle in the realization of a mnemonic BCI to enhance memory formation, and should be the focus of future research. One source of variability is that participants likely have more than one strategy to form memories. This is especially true during free recall in which memories are retrieved using a set of internal memory cues. Because these internal memory cues are unconstrained, a variety of factors during encoding can influence the probability of later free recall. As a result, memory encoding in free recall is very complex (Howard and Kahana, 1999); there is a well-documented encoding advantage for early-list items (Murdock, 1962), late-list items (Postman and Phillips, 1965), items nearby in list position (Kahana, 1996), and items nearby in semantic meaning (Romney et al., 1993).

Behavioral studies have shown that participants use a combination of these encoding strategies to remember the items, and such strategies are antagonistic. For example, the more a person recalls words using a temporal encoding strategy, the less likely they are to use a semantic encoding strategy (Healey et al., 2014). In addition, many of these behavioral effects have different neurophysiological correlates (Sederberg et al., 2006; Long et al., 2010; Staudigl and Hanslmayr, 2013; Serruya et al., 2014). For example, if theta activity reflects a temporal encoding strategy (Staudigl and Hanslmayr, 2013), then reducing theta activity could simply force the individual to rely on semantic encoding strategies, leaving the overall rate of recall intact. This may explain why triggering off of theta power may not impact overall recall ability. Another example of variability is the issue of whether theta increases or decreases predict memory formation. The amount that decreases in theta power actually enhance human memory encoding may ultimately explain much of the variance in these data, and future research should more firmly link how theta oscillations relate to human memory (Hanslmayr and Staudigl, 2014).

Finally, we note that although this study used iEEG to trigger word presentation, we recognize that other studies have found non-invasively recorded MTL theta activity is increased before the presentation of items that are later successfully encoded (Guderian et al., 2009). However, surface recordings are limited in their spatial resolution. Using iEEG, we can identify predictive markers for subsequent encoding with good spatial specificity. Furthermore, depth electrodes enable the identification of pre-stimulus activity within structures such as the hippocampus, which may be a more effective target for enhancing memory function. Future studies would be well-served to explore these possibilities using surface recordings, but it still remains unclear whether the limited spatial resolution offered by these recordings will afford sufficient specificity to predict subsequent memory encoding, and may involve source localization procedures to target markers of encoding with greater fidelity.

In conclusion, here we have linked pre-stimulus theta/alpha oscillations, which have been previously correlated with the ability to encode memories, to the act of forming a memory. If such oscillations play a mechanistic role in encoding, then their presence should boost memory formation. We found that, although theta oscillations were able to improve memory in a few sessions, the result was not consistently observed across all participants. The main utility of this work is that it is the first device, to our knowledge, to use iEEG in a BCI to enhance memory. This provides a proof-of-principle that a BCI driven off of chronically implanted electrodes could serve as a “memory boosting” device.

## Author contributions

John F. Burke, Kareem A. Zaghloul, Joshua Jacobs, and Michael J. Kahana designed research; John F. Burke and Maxwell B. Merkow performed research; John F. Burke, Kareem A. Zaghloul, and Maxwell B. Merkow analyzed data; John F. Burke, Kareem A. Zaghloul, Joshua Jacobs, Maxwell B. Merkow, and Michael J. Kahana wrote the paper.

## Funding

This work was supported by National Institutes of Health grant R21NS067316 and the Dana Foundation.

### Conflict of interest statement

The authors declare that the research was conducted in the absence of any commercial or financial relationships that could be construed as a potential conflict of interest.
