# Supplementary material for: Brain computer interface to enhance episodic memory in human participants
Source: Front Hum Neurosci. 2015 Jan 20;8:1055. doi: 10.3389/fnhum.2014.01055 (PMC4299435; doi:10.3389/fnhum.2014.01055)

Frontiers in Human Neuroscience

Research Article
11 January 2015

Supplementary Material: Brain computer interface to enhance episodic memory in human participants

John F. Burke [1], Maxwell B. Merkow [2], Joshua Jacobs [3] Michael J. Kahana [4,*] and Kareem A. Zaghloul [5,*]

[1] *Perelman School of Medicine, University of Pennsylvania, Philadelphia, PA, USA*
[2] *Department of Neurosurgery, University of Pennsylvania, Philadelphia, PA, USA*
[3] *Biomedical Engineering, Science & Health Systems, Drexel University, Philadelphia, PA, USA*
[4] *Department of Psychology, University of Pennsylvania, Philadelphia, PA, USA*
[5] *Surgical Neurology Branch, NINDS, National Institutes of Health, Bethesda, MD, USA*

Correspondence*:
Kareem A. Zaghloul
Surgical Neurology Branch, NINDS, National Institutes of Health, Bethesda, MA, 20894, USA,
kareem.zaghloul@nih.gov

1 SUPPLEMENTARY TABLES AND FIGURES

The following supplemental information contains two figures and one table. The figures recapitulate the analysis in Figure 2 in the main text, however the results are shown as a function of anatomical region. In the Table, additional information about the patients is given, including the location of the electrodes. The figures and the table are included in the following pages, but the captions are listed below:

Table 1. Electrocorticographic patient population. For each participant, the identification number (**ID**), gender, age, percentage of correctly encoded words (**% Rec**), number of bipolar electrode pairs (**# BPD**), pass-band of the amplifier's filter settings, and a brief anatomical description of the electrode coverage are listed. **TJUH**: Thomas Jefferson University Hospital; **HUP**: Hospital of the University of Pennsylvania; † The electrode montage was changed during the hospital stay. The second montage from patient TJUH 3 contained 81 bipolar derivations.

Supplementary figure 1. Pre–stimulus subsequent memory effect by anatomical lobe. Across all patients, t-statistics (y-axis) comparing spectral power during the presentation of items that were later recalled versus those that were later not-recalled are plotted for all frequencies (x-axis) for the pre-stimulus interval (1000-0 ms before word onset). The data are shown for all electrodes in the anatomical areas of interest, separated by anatomical lobe. The number of patients in each lobe is shown at the top of each plot (**N**). A positive t-statistic represents more power in the recalled versus the not-recalled condition across all 14 patients. The yellow box marks the theta frequency range. The horizontal lines mark the $p = 0.05$ significance level.

Supplementary figure 2. Post–stimulus subsequent memory effect by anatomical lobe. Analogous analysis as in Supplemental Figure 1 for the post-stimulus time window (300-1500 ms after word onset)

1.1 SUPPLEMENTARY TABLE 1

ID	Gender	Age	% Rec	# BPD	pass-band (Hz)	Electrode Coverage
TJUH 1	F	40	17.1	80	0.5 - 100	b/l Depths; b/l Temp Strips
TJUH 2	M	39	22.1	47	0.5 - 100	b/l Depths; b/l Temp/Front Strips
TJUH 3	F	34	22.7	95†	0.5 - 100	b/l Depths; R Temp-Front-Par Strips
TJUH 4	M	33	27.1	105	0.03 - 600	b/lTemp-Front-Par Strips
TJUH 5	F	48	35.0	84	0.03 - 600	b/l Temp-Front-Occip Strips
TJUH 6	M	33	37.6	46	0.03 - 1200	L Temp Strips + STG Depths
TJUH 7	M	23	34.3	78	0.03 - 600	R Depths; R Temp-Front-Par Strips
TJUH 8	M	35	22.8	90	0.03 - 600	b/l Front-Temp Strips; b/l depths
TJUH 9	F	48	20.4	133	0.03 - 600	b/l strips (all lobes); b/l depths
TJUH 10	F	20	40.3	126	0.03 - 600	b/l strips (all lobes); R depths
THUH 11	M	20	29.6	63	0.03 - 600	R Temp-Occip Strips; R depths
THUH 12	M	18	25.6	91	0.03 - 600	b/l Temp-Front Strips; b/l depths
HUP 1	M	37	18.8	123	1.6 - 134	R Temp-Front-Par Grid + Strips
HUP 2	M	42	34.6	55	1.6 - 134	R Temp-Front-Par Grid + R. Temp-Occip grid

1.2 SUPPLEMENTARY FIGURE 1

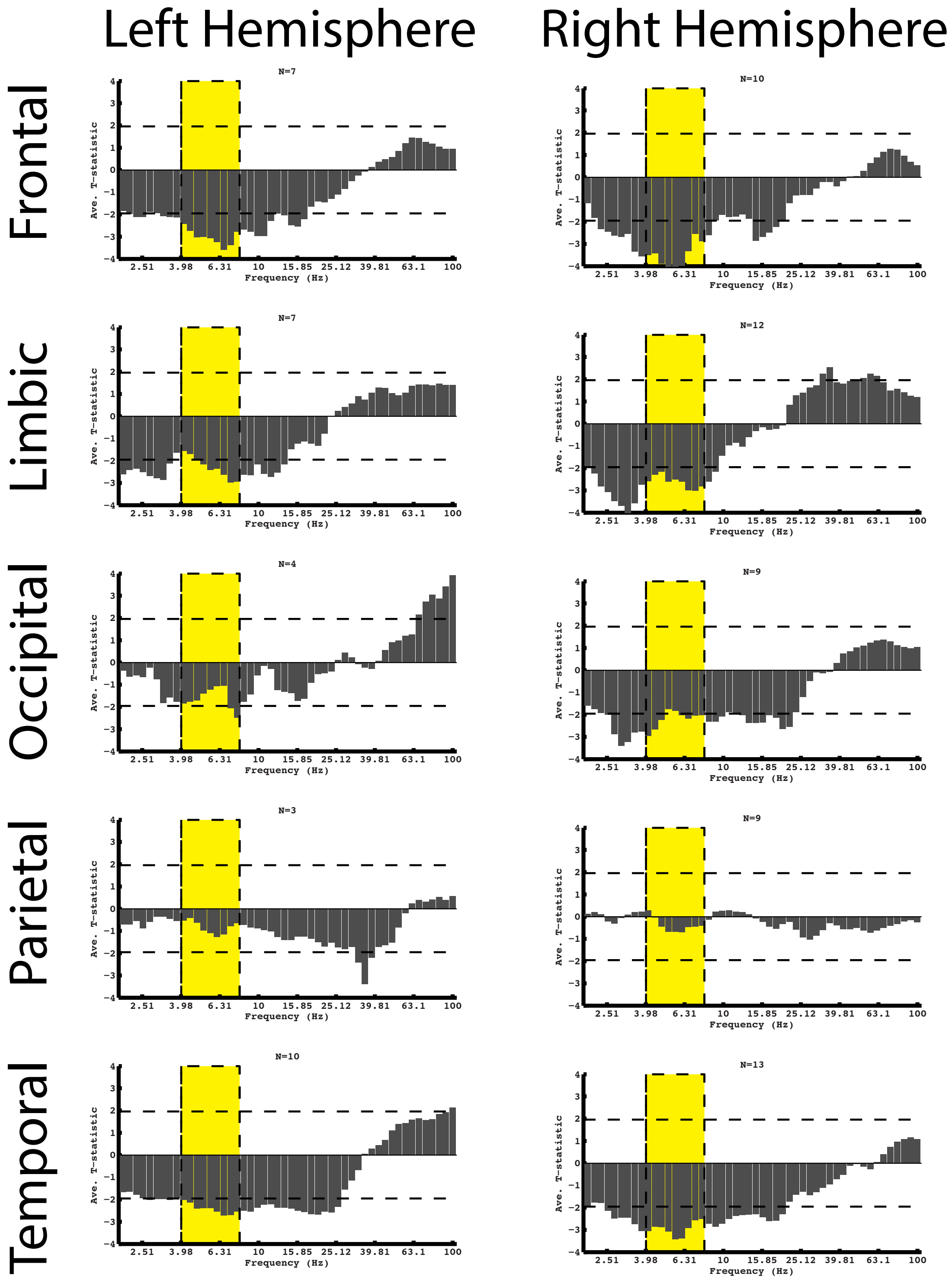

1.3 SUPPLEMENTARY FIGURE 2

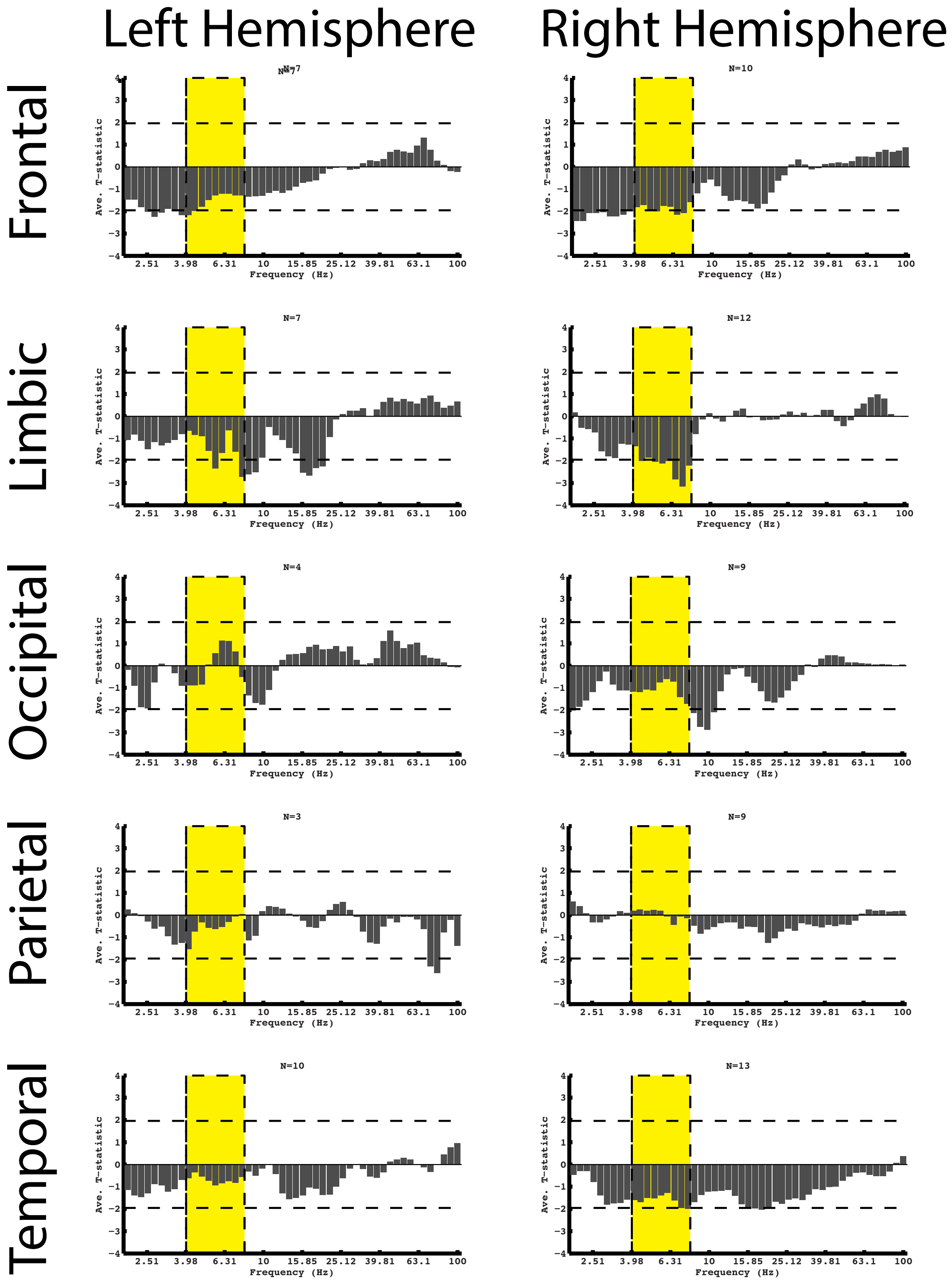

Supplement: Supplementary file 1 [file Presentation1.PDF]
